# Ozone therapy in periodontics

**Published:** 2012-03-05

**Authors:** G Gupta, B Mansi

**Affiliations:** Institute of Dental Studies and Technologies, Uttar Pradesh

**Keywords:** Antimicrobial, Immunostimulating, Antihypoxic, Biosynthetic

## Abstract

Gingival and Periodontal diseases represent a major concern both in dentistry and medicine. The majority of the contributing factors and causes in the etiology of these diseases are reduced or treated with ozone in all its application forms (gas, water, oil). The beneficial biological effects of ozone, its anti-microbial activity, oxidation of bio-molecules precursors and microbial toxins implicated in periodontal diseases and its healing and tissue regeneration properties, make the use of ozone well indicated in all stages of gingival and periodontal diseases. The primary objective of this article is to provide a general review about the clinical applications of ozone in periodontics. The secondary objective is to summarize the available in vitro and in vivo studies in Periodontics in which ozone has been used. This objective would be of importance to future researchers in terms of what has been tried and what the potentials are for the clinical application of ozone in Periodontics.

## Introduction

The word ozone comes from the Greek “ozein” meaning odorant. Ozone (also known as triatomic oxygen and trioxygen) is an allotropic form of oxygen occurring naturally in the Earth’s atmosphere. It surrounds the Earth at an altitude of between 50,000 and 100,000 feet. [**1**] It is created in nature when ultraviolet rays cause oxygen atoms to temporarily recombine in groups of three. It is also formed by the action of electrical discharges on oxygen, so it is often created by thunder and lightning.

It has got the capacity to absorb the harmful ultra-violet rays present in the light spectrum from the Sun. It is a pale blue gas that condenses to a deep blue liquid at very low temperatures.

Ozone is an unstable gas and it quickly gives up nascent Oxygen molecule to form Oxygen gas. Due to the property of releasing nascent Oxygen, it has been used in human medicine since long back to kill bacteria, fungi, to inactivate viruses and to control hemorrhages. [**2**] Medical grade ozone is made from pure medical oxygen. It is produced commercially in ozone generators, which involves sending an electrical discharge through a specially-built condenser containing oxygen. 

### History

First discovered until 1840 by the German chemist Christian Frederick Schonbein at the University of Basil in Switzerland, ozone was first used in medicine in 1870 by Landler. However, it was not until 1932 that ozone was seriously studied by the scientific community, when ozonated water was used as a disinfectant by Dr. E. A. Fisch, [**3**] a Swiss dentist. Fisch had the first idea to use ozone as either a gas or ozonated water in his practice. By a twist of fate, a surgeon, Dr. E Payr (1871–1946) had to be treated for a gangrenous pulpite and remained astonished by the result achieved with local ozone treatment. He enthusiastically extended its application to general surgery.

At the time, ozone therapy was difficult and limited due to the lack of ozone-resistant materials, such as Nylon, Dacron, and Teflon, until 1950 when ozone-resistant materials were manufactured. At that time Joachim Hänsler, a German physicist and physician, joined another German physician, Hans Wolff, to develop the first ozone generator for medical use. Their design continues to be the basis for modern equipment.

### Ozone generators

There are three different systems for generating ozone gas: [**4**]

• **Ultraviolet System: **produces low concentrations of ozone, used in esthetics, saunas, and for air purification.

• **Cold Plasma System: **used in air and water purification.

• **Corona Discharge System: **produces high concentrations of ozone. It is the most common system used in the medical/ dental field. It is easy to handle and it has a controlled ozone production rate.

Medical grade ozone is a mixture of pure oxygen and pure ozone in the ratio of 0.05% to 5% of ₃ and 95% to 99.95% of O₂. Due to the instability of the ₃ molecule, medical grade ozone must be prepared immediately before use. Within less than an hour after preparation only half of the mixture is still ozone while the other half is transformed into oxygen. As a result, it is impossible to store ozone over long periods of time. In order to control the decomposition of ₃ into oxygen it can be associated with a vehicle with aqueous properties to promote the conversion more quickly or with a vehicle with more viscous properties to retard the conversion.

### Use of ozone in dentistry

The use of ozone in dentistry is gaining its place in every day's dental practice and is used in almost all dental applications. The undisputed disinfection power of ozone over other antiseptics makes the use of ozone in dentistry a very good alternative and/or an additional disinfectant to standard antiseptics. 

Due to safety concerns, ₃ gas was not recommended for intra-oral use. Only dissolved ozone in water and ozonated oils were and are still commonly used in different fields of dentistry. With the development of a foot pedal-activated dental handpiece with a suction feature, ₃ gas can now be used safely in situations where diffusion is an important factor, i.e. dental hard tissues.

According to German dentist Fritz Kramer, [**5**] ozone, such as in the form of ozonated water, can be used in the following ways. 

1. as a powerful disinfectant

2. in its ability to control bleeding

3. in its ability to cleanse wounds in bones and soft tissues. 

4. by increasing the local supply of oxygen to the wound area, ozone can improve healing. 

5. ozonated water can increase temperature in the area of the wound, and this increase the metabolic processes related to wound healing. 

Dr. Kramer points out that ozonated water can be used in a number of different ways:

1. as a mouth rinse (especially in cases of gingivitis, paradentosis, thrush or stomatitis);

2. as a spray to cleanse the affected area, and to disinfect oral mucosa, cavities and in general dental surgery;

3. as an ozone/water jet to clean cavities of teeth being capped, receiving root canal therapy, and in treating painful gingivitis and stomatitis. 

### Biological actions

The application of ozone in dentistry comes as a result of physico-chemical properties: There are several known actions of ozone on human body, such as immunostimulating and analgesic, antihypoxic and detoxicating, antimicrobial, bioenergetic and biosynthetic (activation of the metabolism of carbohydrates, proteins, lipids) etc. [**6**]

**1. Antimicrobial effect**- Ozone works destructively against bacteria, fungi, and viruses. The antimicrobial effect of ozone is a result of its action on cells by damaging its cytoplasmic membrane due to ozonolysis of dual bonds and also ozone-induced modification of intracellular contents (oxidation of proteins loss of organelle function) because of secondary oxidants effects. This action is non-specific and selective to microbial cells; it does not damage human body cells because of their major antioxidative ability. Ozone is very efficient in antibiotics resistant strains. Its antimicrobial activity increases in liquid environment of the acidic pH. In viral infections the ozone action lies in the intolerance of infected cells to peroxides and change of activity of reverse transcriptase, which takes part in synthesis of viral proteins. [**6**] Being a very strong oxidant it joins with biomolecules containing cysterine, cysteine, methionine, histidine (all being part of bacterial cell membranes. The main targets of their attack are the thiol groups of the amino acid cysteine. As a result of the reaction of ozone with unsaturated fatty acids of a lipid sheath of a virus the lipid sheath of a virus melts. The research shows that a few-second-application of ozone stops all vital functions of bacteria which are incapable of developing any self-immunity to its action. Gram+ (Gram-positive) bacteria are more sensitive to the action of ozone than Gram– (Gram-negative) bacteria. Oxygen-free bacteria react to ozone as well. Among cariogenic bacteria *Streptococcus mutans* and *Streptococcus sobrinus* are the most sensitive. Ozone easily acts on multi unsaturated fatty acids which occur in virus sheaths. Ozone reacts also with ascorbinians and tocopherols. [**7**]

**2. Immunostimulating Effect**- Ozone influences cellular and humoral immune system. It stimulates proliferation of immunocompetent cells and synthesis of immunoglobulins. It also activates function of macrophages and increases sensitivity of micro-organisms to phagocytosis. [**6**] As a response to this activation through ozone, the body's immune cells produce special messengers called cytokines. These molecules in turn activate other immune cells, setting off a cascade of positive change throughout the immune system, which is stimulated to resist diseases. This means that the application of medical ozone is extremely useful for immune activation in patients with a low immune status and/or immune deficit. [**8**] Ozone causes the synthesis of biologically active substances such as interleukins, leukotrienes and prostaglandins which is beneficial in reducing inflammation and wound healing. [**6**] Ozone in high concentration causes immunodepressive effect whereas in its low concentration immunostimulating effect. [**7**]

**3. Antihypoxic effect**- Ozone brings about the rise of pO2 in tissues and improves transportation of oxygen in blood, which results in change of cellular metabolism – activation of aerobic processes (glycolysis, Krebs cycle, β-oxidation of fatty acids) and use of energetic resources. Repeating low doses of ozone activate enzymes: super-oxide dismutases, catalases, dehydrogenase, and glutatione peroxidases. They are part of complex enzymatic systems which protect organisms against the action of oxygen-free radicals. It also prevents formation of erythrocytes aggregates and increases their contact surface for oxygen transportation. Its ability to stimulate the circulation is used in the treatment of circulatory disorders and makes it valuable in the revitalizing organic functions. [**6**] Ozone improves the metabolism of inflamed tissues by increasing their oxygenation and reducing local inflammatory processes. By changing the cell membrane structure of erythrocytes and causing the increase of its negative charge it influences the structure change as well as blood cell elasticity. This in consequence reduces blood cell rolling and enables blood flow in capillary vessels. By increasing the concentration of 2,3 Diphosphoglycerate (2,3-DPG), ozone changes the configuration of erythrocytes, which enables them to return oxygen in the inflamed tissue. [**7**]

**4. Biosynthetic Effect**- It activates mechanisms of protein synthesis, increases amount of ribosomes and mitochondria in cells. These changes on the cellular level explain elevation of functional activity and regeneration potential of tissues and organs. [**6**]

5. Ozone causes secretion of vasodilators such as NO, which is responsible for dilatation of arterioles and venules. [**6**] It also activates angiogenesis. [7]

6. Ozone, when acting on the organic substance of mineralized tooth tissues intensifies their remineralization potential. At the same time, it is capable of “opening” dentinal tubules, which enables the diffusion of calcium and phosphorus ions to the deeper layers of carious cavities. [**9**]

A high concentration of ozone kills bacteria very quickly and is thousand times more powerful than other bacterial killing agents. The average concentration of ozone used in treatments is 25 gm of ozone per milliliter of oxygen/ozone gas mixture that translates into 0.25 parts of ozone to 99.75 parts of oxygen. Evidence-based research has shown that at this concentration, ozone effectively kills bacteria, fungi, viruses and parasites. [**10**] As an antimicrobial agent, it is a powerful oxidizer at a dramatically lower concentration than chlorine with none of the toxic side effects. One molecule of ozone is equal to between 3,000 to 10,000 molecules of chlorine and it kills pathogenic organisms 3,500 times faster. [**10**] Studies have revealed that it only takes 10 sec to kill 99 % of bacteria, fungi and viruses. [**11**] It can oxidize many organic compounds and it is a powerful germicide. [**12**] Some of the other effects are circulatory enhancement, disruption of tumor metabolism and stimulation of oxygen metabolism. [**13**]

According to most authors, a 10-sec-application of ozone causes the destruction of 99% of bacteria, and a 20-sec-application even of 99.9%. In this way, so-called ecological niche appears.

However, it is not conducive to their repeated colonization within 4 to 6 weeks. [**14,15**] Ozone is not toxic when it is given in the amount of 0.05 ppm for 8 hours. During ozone therapy a maximum concentration of ozone in oral cavity amounts to 0.01 ppm.

### Goals of ozone therapy

Setting the standard-of-care and therapeutic goals are based on sound evidence-based science is critical. Therapeutic goals are inclusive and not exclusive of standard of care. The goals of oxygen/ozone therapy are: [**10**]

1. Elimination of pathogens.

2. Restoration of proper oxygen metabolism.

3. Induction of a friendly ecologic environment.

4. Increased circulation.

5. Immune activation.

6. Simulation of the humoral anti-oxidant system.

### Use in dentistry

**Periodontology**- Gingivitis, Periodontits, Periimplantitis, Surgical cuts, Prophylaxis

**Dental and oral pathology**- Caries, Enamel cracks, Root canal treatment, Tooth whitening, Dentinal hypersensitivity, Abscess, Granuloma, Fistulae, Apthae, Herpes infection, Stomatitis – Candidiasis

**Surgery**- Implantation, Re-plantation, Extraction, Wound Healing, Coaguloapathy - prolonged bleeding

**Prosthodontics and restorative dentistry**- Stumps and crown disinfection, Cavity disinfection

**Orthodontics and orthopedics**- TMJ dysfunctions, Trismus, Relaxation, Myoarthtopathy

**Diagnostics**- Vitality test

### Appliances producing ozone for dental use

**1. HealOzone by KaVo** is air-based and the application of the gas takes place in a closed circuit. Its surplus is sucked out and neutralized by manganese ions. The concentration of ozone in the cap adjacent to the tissue amounts to 2100 ppm. Perfect air tightness of the cap is necessary for the application of ozone. Therefore, the application is only possible on the surfaces where such air tightness can be provided.

**2. OzonyTron by MYMED Gmb H.** - Oxygen activation generator (OzonytronX—Biozonix, München, Germany) uses the power of high frequency and voltage. Activated oxygen (ozone) concentration can be adjusted in 5 levels via current strength. Inside the glass probe, which is formed by a double glass camera, is a noble gasses mixture that is conducting and emitting electromagnetic energy. When the tip of the probe gets in contact with the body it emits energy around the treated area and splits environmental diatomic oxygen in singular atomic oxygen and ozone. The concentration of ozone in the operation field is 10 to 100 μg/ml (becomes a fungi-, viru-, and bacteriocide at the intensity of 1–5 μg/ml). There is no closed circuit here, therefore, ozone can be applied to the places that are difficult to reach, e.g. gingival pockets or root canals.

**3. Product photo (Prozone) by W&H** - It is characterized by its ease of use and safety of application (preset tissue-compatible dosages in the indication areas of periodontitis and endodontitis). Prozone ensures a hygienic procedure during the gassing of the pockets due to its exchangeable plastic attachments (Perio tips or Endo tips).

### Route of ozone administration

**1. Gaseous Ozone** - Gaseous ozone is most frequently used in restorative dentistry and endodontics. Topical administration of the gaseous form can be via an open system or via a sealing suction system as a prerequisite to avoid inhalation and adverse effects. Ozone appears to be an integral part of noninvasive therapy of dental caries, as a disinfectant prior to placing a direct restoration and as therapy for hypomineralized teeth. [**16**]

**2. Ozonated Water** - Ozonated water has been shown to be very effective against bacteria, fungi and viruses and is also less expensive compared to other chemical cleaners. [**17**] Gaseous ozone was shown to be a more effective microbicide than the aqueous form and, applied for 3 min, may be used as a dental disinfectant. [**18**] Because ozone gas has been found to have toxic effects if inhaled into the respiratory tract, [**16,18,19**] ozonated water may be useful to control oral infections and various pathogens.[15] 

**3. Ozonized Oil** - In addition to ozone application in its gaseous and aqueous form, sunﬂower ozonized oil also seems extremely convenient. The wide accessibility of sunﬂower oil makes it a competitive antimicrobial agent. Ozonized oil (Oleozone, Bioperoxoil) has shown to be effective against Staphylococci, Streptococci, Enterococci, Pseudomonas, Escherichia coli and especially Mycobacteria [**16,20,21**] and has been utilized for the cure of fungal infections. [**16,20**] 

### Ozone therapy in periodontics

The main use of ozone in dentistry is relays on its antimicrobial properties. It is proved to be effective against both Gram positive and Gram negative bacteria, viruses and fungi. [**22**] 

Ebensberger et al [**23**] evaluated the effect of irrigation with ozonated water on the proliferation of cells in the periodontal ligament adhering to the root surfaces of 23 freshly extracted completely erupted third molars. The teeth were randomly treated by intensive irrigation with ozonated water for 2 min or irrigation with a sterile isotonic saline solution, serving as a control group. The periodontal cells of these teeth were studied immunohistochemically to mark proliferating cell nuclear antigen (PCNA). It was observed that the labeling index (the number of positive cells compared to the total number of cells suggesting enhancement of metabolism) was higher among the teeth irrigated with ozone (7.8% vs. 6.6%); however, the difference was not statistically significant (p = 0.24). They concluded that the 2 min irrigation of the avulsed teeth with non-isotonic ozonated water might lead not only to a mechanical cleansing, but also decontaminate the root surface, with no negative effect on periodontal cells remaining on the tooth surface.

Nagayoshi et al [**24**] examined the effect of ozonated water on oral microorganisms and dental plaque. Dental plaque samples were treated with 4mL of ozonated water for 10 s. they observed that ozonated water was effective for killing gram-positive and gram-negative oral microorganisms and oral Candida albicans in pure culture as well as bacteria in plaque biofilm and therefore might be useful to control oral infectious microorganisms in dental plaque.

Nagayoshi et al [**17**] tested the efficacy of three different concentrations of ozone water (0.5, 2, and 4 mg/ml in distilled water) on the time-dependent inactivation of cariogenic, periodontopathogenic and endodontopathogenic microbes (Streptococcus, Porphyromonas gingivalis and endodontalis, Actinomyces actinomycetemcomitans, Candida albicans) in culture and in biofilms. They confirm that ozonated water was highly effective in killing of both gram positive and gram negative micro-organisms.

Depending on the dosage, the oral microbes were inactivated after 10 seconds. Gram negative anaerobes, such as Porphyromonas endodontalis and Porphyromonas gingivalis were substantially more sensitive to ozonated water than gram positive oral streptococci and Candida albicans in pure culture. Furthermore ozonated water had strong bactericidal activity against bacteria in plaque biofilm. In addition, ozonated water inhibited the accumulation of experimental dental plaque in vitro.

Ramzy et al [**24**] irrigated the periodontal pockets by ozonized water in 22 patients suffering from aggressive periodontitis (age range from 13 to 25 years). Periodontal pockets were irrigated with 150 ml of ozonized water over 5 to 10 minutes once weekly, for a clinical four weeks study, using a blunt tipped sterile plastic syringe. High significant improvement regarding pocket depth, plaque index, gingival index and bacterial count was recorded related to quadrants treated by scaling and root planning together with ozone application. They also reported significant reduction in bacterial count in sites treated with ozonized water.

Huth et al [**19**] in their study declared that the aqueous form of ozone, as a potential antiseptic agent, showed less cytotoxicity than gaseous ozone or established antimicrobials (chlorhexidine digluconate-CHX 2%, 0.2%; sodium hypochlorite-NaOCl 5.25%, 2.25%; hydrogen peroxide-H₂O₂ 3%) under most conditions. Therefore, aqueous ozone fulfils optimal cell biological characteristics in terms of biocompatibility for oral application.

Huth et al [**26**] in their later paper examined the effect of ozone on the influence on the host immune response. These researchers chose the NF-kappaB system, a paradigm for inflammation-associated signalling/transcription. Their results showed that that NF-kappaB activity in oral cells in periodontal ligament tissue from root surfaces of periodontally damaged teeth was inhibited following incubation with ozonized medium. The Huth 2007 study establishes a condition under which aqueous ozone exerts inhibitory effects on the NF-kappaB system, suggesting that it has an anti-inflammatory capacity. 

Muller et al [**27**] compared the influence of ozone gas with photodynamic therapy (PDT) and known antiseptic agents (2% Chlorhexidine, 0,5 and 5% hypochlorate solutions) on a multispecies oral biofilm in vitro. The following bacteria were studied – Actinomyces naeslundii, Veillonella dispar, Fusobacterium nucleatum, Streptococcus sobrinus, Streptococcus oralis and Candida albicans. Gasiform ozone was produced by vacuum ozone delivery system Kavo Healozone. They concluded that the matrix-embedded microbial populations in biofilm are well protected towards antimicrobial agents. Only 5 % Hypochlorate solution was able to eliminate all bacteria effectively. Usage of gasiform ozone or PDT was not able to reduce significantly or completely eliminate bacteria in the biofilm.

Kronusová [**28**] used ozone in following cases: prevention of dental caries in fissures of the first permanent molars in children, application of ozone in prepared cavity, after tooth extraction, in case of postextractional complications, in patients with chronic gingivitis, periodontitis and periodontal abscesses, herpes labialis, purulent periodontitis, dentition difficilis, etc. Almost all patients with gingivitis showed subjective and objective improvement of their status, as well as patients with periodontal abscess, where no exsudation was observed. Application of ozone after tooth extraction was found also quite useful – only 10 % of patients suffered from such complication as alveolitis sicca, but even in these cases the clinical course was shorter and more moderate.

The influence of ozonized water on the epithelial wound healing process in the oral cavity was observed by Filippi. [**29**] It was found that ozonized water applied on the daily basis can accelerate the healing rate in oral mucosa. This effect can be seen in the first two postoperative days. The comparison with wounds without treatment shows that daily treatment with ozonized water accelerates the physiological healing rate.

In the study by Karapetian et al, [**30**] periimplantitis treatment with conventional, surgical and ozone therapy methods was investigated, and it was found that the most effective bacteria reduction was in the ozone-treated patient group. The authors concluded that the main challenge seems to be the decontamination of the implant surface, its surrounding tissue and the prevention of recolonization with periodontal pathogenic bacteria. 

Kshitish and Laxman [**31**] conducted a randomized, double-blind, crossover split-mouth study on 16 patients suffering from generalized chronic periodontitis. The study period of 18 days was divided into two time-intervals, i.e. baseline (0 days) to 7th day, with a washout period of 4 days followed by a second time interval of 7 days.

Subgingival irrigation of each half of the mouth with either ozone or chlorhexidine was done at different time intervals. They observed a higher percentage of reduction in plaque index (12%), gingival index (29%) and bleeding index (26%) using ozone irrigation as compared to chlorhexidine. The percentile reduction of Aa (25%) using ozone was appreciable as compared to no change in Aa occurrence using chlorhexidine. By using ₃ and chlorhexidine, there was no antibacterial effect on *Porphyromonas gingivalis* (Pg) and *Tannerella forsythensis*. The antifungal effect of ozone from baseline (37%) to 7th day (12.5%) was pronounced during the study period, unlike CHX, which did not demonstrate any antifungal effect. No antiviral property of ozone was observed. The antiviral efficacy of chlorhexidine was better than that of ozone. They concluded that despite the substantivity of chlorhexidine, the single irrigation of ozone is quite effective to inactivate microorganisms.

### Application modalities

According to the clinical case, different applications modalities are available using ozone gas, irrigation with ozonated water and in-office use of ozonized oil as well as home use.

**Gas application via a customized thermoformed dental appliance**- A customized suckdown thermoformed hard or medium-soft dental appliance can be prepared. It should extend 2-3 mm beyond the affected gingival area, leaving a free space for gas circulation. 2 ports should be attached for the gas inlet and outlet respectively at the distal and mesial of the treatment area. The edges of the appliance should be reclined with light or medium body silicone. Light-cured dam can also be applied as an extra safety precaution to completely seal the borders. The ports to the generator and the suction pump should then be attached. This procedure will treat both hard and soft tissues of the affected area. PVC or silicone cap can be used to treat individually all the indicated areas in difficult situations where such an appliance is hard to use or uncomfortable to the patient.

**Irrigation with Ozonated Water**- Ozonated water can be used to irrigate the affected area during and after scaling, root surface planning, and non-surgical pocket curettage.

**In-office and Home Use of Ozonized Olive Oil**- After in-office treatment with ozone gas or ozonated water, pockets can be filled with ozonized olive oil using a blunt 25G needle or any other appropriate tip. Patient can be given some of the oils for home use. In-office ozonized oil application can be repeated once a week.

**Surgical Procedures**- Ozonated water can be used as an irrigant during the surgical procedure and/or as a final surgical site lavage. The sutures can be covered with a thin layer of ozonized oil and the patient can be instructed to apply the oil 3-4 times a day.

**Peri-Implantitis**- Peri-implantitis is very bothering to both the dentist and the patient. After thorough assessment and if a decision is taken to salvage the case, different modes of therapy are used in order to save the implant from total loss. Laser and/or manual debridement along with antiseptic solutions and topical anti-microbial medicines are commonly performed with a varying degree of success. Ozone can play an important role and be used as gas or in aqueous form. An appropriate length of PVC or silicone cap can be cut to cover the abutment fully. It should properly seal the gingival borders around the implant. Ozone gas infiltrations can also be used in this situation. Ozonated water can be used as an irrigant during debridement and curettage. Patient can be advised to apply ozonized oil on the treated area 3-4 times/day. 

### Desensitization of sensitive root necks [2]

Quick and prompt relief from root sensitivity has been documented after ozone spray for 60 seconds followed by mineral wash onto the exposed dentine in a repetitive manner. This desensitization of dentine lasts for longer period of time. Smear layer present over the exposed root surface prevents the penetration of ionic Calcium and Fluorine deep into the dentinal tubules. Ozone removes this smear layer, opens up the dentinal tubules, broadens their diameter and then Calcium and Fluoride ions flow into the tubules easily, deeply and effectively to plug the dentinal tubules, preventing the fluid exchange through these tubules. Thus, ozone can effectively terminate the root sensitivity problem within seconds and also lasts longer than those by conventional methods.

### Ozone toxicity

Ozone inhalation can be toxic to the pulmonary system and other organs. Complications caused by ozone therapy are infrequent at 0.0007 per application. Known side-effects are epiphora, upper respiratory irritation, rhinitis, cough, headache, occasional nausea, vomiting, shortness of breath, blood vessel swelling, poor circulation, heart problems and at a times stroke. [**44**] Because of ozone's high oxidative power, all materials that come in contact with the gas must be ozone resistant, such as glass, silicon, and Teflon. However, in the event of ozone intoxication the patient must be placed in the supine position, and treated with vitamin E and n-acetylcysteine. [**16**]

### Contraindications

The following are contraindications for use of ozone therapy: [**4**]

• Pregnancy

• Glucose-6-phosphate-dehydrogenase deficiency (favism)

• Hyperthyroidism

• Severe anaemia

• Severe myasthenia

• Active hemorrhage

• Acute alcohol intoxication

• Recent Myocardial infarction

## Discussion

Gingivitis and periodontitis are characterized by a local hypoxia of tissues and also by various microbic florae that may contain over 500 species. Accumulations of bacterial plaque in the gingival crevice area in an increased amount causes changes in the oral cavity ecology leading to both gingivitis and periodontitis. [**33**] 

Dental biofilm makes it difficult for antibiotics in targeting putative periodontal pathogens. Higher concentrations of antibiotics are required to kill these organisms which are inevitably associated with toxic adverse effect on the host microbial flora. The application of ozone therapy in periodontics showed promising results. Both gaseous and aqueous ozone are used as a substitute to mechanical debridement. Ozone can be used to help treat periodontal disease by using ozonated water flushed below the gum line and/or ozone gas infiltrated into the gum tissue and supporting tissues. 

Ozonated water (4mg/l) strongly inhibited the formation of dental plaque and reduced the number of sub gingival pathogens both gram positive and gram negative organisms. Gram negative bacteria, such as P. endodontalis and P. gingivalis were substantially more sensitive to ozonated water than gram positive oral streptococci and C. albicans in pure culture. [**17**] Furthermore ozonated water had strong bactericidal activity against bacteria in plaque biofilm. In addition, ozonated water inhibited the accumulation of experimental dental plaque in vitro. [**17**] 

Dental researchers have started to examine the effects of ozonated fluids in periodontal disease. Huth et al in two papers in 2006 [19] and 2007 [**26**] examined the effect of ozone on periodontal tissues. The 2007 paper compared traditional periodontal anti-microbial products with the use of ozonated water. Both papers concluded that ozonated water has an excellent anti-microbial effect. 

It resulted in toxic effect on human oral epithelial and fibroblast cells compared to antiseptics such as chlorehexidine digluconate, sodium hypochlorite and hydrogen peroxide during a 1-minute time period. [**34,35**] Ozone gas found to be toxic to the human oral epithelial and gingival fibroblast cells and aqueous ozone was more biocompatible than gaseous ozone. [**19**] The application of ozone therapy in chronic gingival and periodontal diseases, showed subjective and objective improvement of their status, as well as patients with periodontal abscess, with no exudation was observed. [**36**]

Brauner [**37**] has demonstrated that the combination of professional tooth cleaning and daily rinsing of the mouth with ozone water can improve clinical findings in cases of gingivitis and periodontitis. Plaque indices and a tendency to bleed, however, quickly return if the professional measures are interrupted. Rinsing the mouth with ozone water without any mechanical procedures for plaque reduction were unsuccessful.

There are many benefits to control oral hygiene and as a source of sterile water. However, patients should also be informed that there is an interaction of aqueous ozone with anti-microbials. This research has been published, illustrating the importance of potential interactions of dissolved ozone and prescribed anti-microbials. Patients who are taking a course of antibiotics may need to be informed that the use of ozonated water inactivates antibacterial agents [**28**] and in particular amoxicillin, [**29**] progesterone [**30**] and tetracycline. [**31**] What concerns the dentists is that ozone may inactivate the anti-microbial effects of triclosan. [**32**]

The effect of ozone on wounds obtained in the process of surgical and implantological procedures is used to prevent complications like after-surgical infection and to conduce to proper tissue healing. The use of ozone around implants is supported by published research showing that ozone not only effectively sterilizes the surfaces of both the implant and bone, but also switches on the reparative mechanisms allowing tissue regeneration around implant surfaces. [**43**] According to Matsumura et al [**44,45**] ozone does not have a major impact on stimulation of gingival cells for osteoblastic activity in the regeneration of the periodontium around implants.

## Conclusions

Dentistry is changing as we are now using modern science to practice dentistry. In comparison with classical medicine modalities such as antibiotics and disinfectants, ozone therapy is quite inexpensive, predictable and conservative. The ozone therapy has been more beneficial than present conventional therapeutic modalities. This state of the art technology allows us to take a minimally invasive and conservative approach to dental treatment. The elucidation of molecular mechanisms of ozone further benefits practical application in dentistry. Treating patients with ozone therapy reduces the treatment time with a great deal of difference and it eliminates the bacterial count more precisely. The treatment is completely painless and increases the patients' acceptability and compliance with minimal adverse effects. Although more clinical research has to be done to standardize indications and treatment procedures of ozone therapy, still many different approaches are so promising, or already established, that hopefully the use of ozone therapy becomes a standard treatment for disinfection of an operation sites in dentistry.

## References

[1] (1968). Chemical technology an encyclopedic treatment. Vol 1.

[2] Garg  R, Tandon  S (2009). Ozone: A new face of dentistry.. The Internet Journal of Dental Science.

[3] Fish E, Ophthalmic Ventures (1936). Apparatus for the production and use of ozone in therapeutics. United States Patent.

[4] Nogales  CG, Ferrari  PA, Kantorovich  EO (2008). Ozone Therapy in Medicine and Dentistry. J Contemp Dent Pract.

[5] Krammer  F (1983). Ozone in the dental practice.

[6] Seidler  V, Linetskiy  I, Hubalkova  H (2008). Ozone and Its Usage in General Medicine and Dentistry. A Review Article. Prague Medical Report.

[7] Teresa  B, Wolanska  E, Cieszko-Buk M (2008).

[8] Seaverson  K, Tschetter  D, Kaur  T Patient guide to oxygen/ozone therapy.. Health centered cosmetic dentistry. [Online].

[9] Lynch  E (2004). Leczenie próchnicy z wykorzystaniem systemu HealOzon. eDentico.

[10] Mollica  P, Harris  R Integrating oxygen/ ozone therapy into your practice. [Online]..

[11] Ozone dental therapy. [Online]..

[12] Gum GP and Bone Regeneration Procedures. Australian Institute of Implant Dentistry [online].

[13] Ozone Therapy Oxygen Therapies. Applied ozone systems. [Online].

[14] Baysan  A, Whiley  RA, Lynch  E (2000). Antimicrobial effect of novel ozone generating device on microorganisms associated with primary root carious lesions in vitro. Caries Research.

[15] Holmes  J (2004). Zastosowanie ozonu do leczenia pierwotnych zmian próchnicowych bruzd. Por. Stomat.

[16] Nogales  CG, Ferrari  PH, Kantorovich  EO (2008). Ozone therapy in medicine and dentistry. J Contemp Dental Pract.

[17] Nagayoshi  M, Kitamura  C, Fukuizumi  T (2004). Antimicrobial effect of ozonated water on bacteria invading dentinal tubules. J Endod.

[18] Azarpazhooh  A, Limeback  H (2008). The application of ozone in dentistry: a systematic review of literature. J Dentist.

[19] Huth  KC, Jakob  FM, Saugel  B (2006). Effect of ozone on oral cells compared with established antimicrobials. Eur J Oral Sci.

[20] Sechi  LA, Lezcano  I, Nunez  N (2001). Antibacterial activity of ozonized sunﬂower oil (Oleozon).. J Microbiol.

[21] Rodrigues  KL, Cardoso  CC, Caputo  LR (2004). Cicatrizing and antimicrobial properties of an ozonised oil from sunﬂower seeds. Inﬂammopharmacology.

[22] Greene  AK, Few  BK, Serafini  JC (1993). A comparison of ozonation and chlorination for the disinfection of stainless steel surfaces. J. Dairy Sci.

[23] Ebensberger  U, Pohl  Y, Filippi  A (2002). PCNA-expression of cementoblasts and fibroblasts on the root surface after extraoral rinsing for decontamination. Dental Traumatology.

[24] Nagayoshi  M, Fukuizumi  T, Kitamura  C (2004). Efficacy of ozone on survival and permeability of oral microorganisms. Oral Microbiology & Immunology.

[25] Ramzy  MI, Gomaa  HE, Mostafa  MI (2005). Management of Aggressive Periodontitis Using Ozonized Water. Egypt. Med. J. N R C.

[26] Huth , Saugel , Jakob  (2007). Effect of aqueous ozone on the NF-kappaB system. J Dent Res.

[27] Muller  P, Guggenheim  B, Schmidlin  PR (2007). Efficacy of gasiform ozone and photodynamic therapy on a multispecies oral biofilm in vitro. Eur. J. Oral. Sci.

[28] Kronusova  M (2007). Aplikace atomarniho kysliku v ordinaci praktickeho zubniho lekaře. Progresdent.

[29] Fillipi A The influence of ozonised water on the epithelial wound healing process in the oral cavity. Clinic of Oral Surgery. Radiology and Oral Medicine, University of Basel, Switzerldand.

[30] Karapetian  VE, Neugebauer  J, Clausnitzer  CE Comparison of Different Periimplantitis Treatment Methods. http://www.helbo.at/datasheets/poster_karapetian_0304.pdf.

[31] Kshitish  D, Laxman  VK (2010). The use of ozonated water and 0.2% chlorhexidine in the treatment of periodontitis patients: A clinical and microbiologic study. Indian J Dent Res.

[32] Ozone Therapy. Diseases and conditions, complementary and alternative medicine.

[33] Nobuhiro  T, Kazuko  Y, Tao  H Effect of initial periodontal therapy on the frequency of detecting Bacteroides forsythus, Porphyromonas gingivalis and Actinobacillus actinomycetemcomitans..

[34] Sorokina  S, Lukinych  L (1997). Ozone Therapy as a Part of a Complex Treatment of a Paradontium Disease.

[35] Nagayoshi  M, Kitamura  C, Fukuzumi  T (2004). Antimicrobial effect of ozonated water on bacteria invading dentinal tubules. J Endod.

[36] Kronusova  M (2007). Aplikace atomarniho kysliku v ordinaci praktickeho zubniho lekaře. Progresdent.

[37] Brauner  A (1991). Klinische Untersuchung über den therapeutischen Erfolg von ozonisiertem Wasser bei Gingivitis und Parodontitis. [A clinical investigation of the therapeutic success of ozonized water in treating gingivitis and periodontitis]. Zahnärztl. Praxis.

[38] Dodd  MC, Buffle  MO, Von Gunten  U (2006). Oxidation of antibacterial molecules by aqueous ozone: moiety-specific reaction kinetics and application to ozone-based wastewater treatment. Environ Sci Technol.

[39] Andreozzi  R, Canterino  M, Marotta  R (2005). Antibiotic removal from wastewaters: the ozonation of amoxicillin. J Hazard Mater.

[40] Barron  E, Deborde  M, Rabouan  S (2006). Kinetic and mechanistic investigations of progesterone reaction with ozone. Water Res.

[41] Dalmázio  I, Almeida  MO, Augusti  R (2007). Monitoring the degradation of tetracycline by ozone in aqueous medium via atmospheric pressure ionization mass spectrometry. J Am Soc Mass Spectrom.

[42] Suarez  S, Dodd  MC, Omil  F (2007). Kinetics of triclosan oxidation by aqueous ozone and consequent loss of antibacterial activity: relevance to municipal wastewater ozonation. Water Res.

[43] Low  SP, Williams  KA, Canham  LT (2006). Evaluation of mammalian cell adhesion on surface-modified porous silicon. Biomaterials.

[44] Matsumura  K, Hyon  SH, Nakajima  N (2004). Surface modiﬁcation of polyethylene-co-vinyl alcohol hydroxyapatite immobilization and control of periodontal ligament cells differentiation. Biomaterials.

[45] Matsumura  K, Hyon  SH, Nakajima  N (2007). Effects on gingival cells of hydroxyapatite immobilized on polyethylene-co-vinyl alcohol. J Biomed Mater Res A.

